# Patients with Alzheimer’s disease have an increased removal rate of soluble beta-amyloid-42

**DOI:** 10.1371/journal.pone.0276933

**Published:** 2022-10-31

**Authors:** Dmitry V. Zaretsky, Maria V. Zaretskaia, Yaroslav I. Molkov, for the Alzheimer’s Disease Neuroimaging Initiative

**Affiliations:** 1 Zarbio, Chapel Hill, NC, United States of America; 2 Department of Mathematics and Statistics and Neuroscience Institute, Georgia State University, Atlanta, GA, United States of America; Nathan S Kline Institute, UNITED STATES

## Abstract

Senile plaques, which are mostly composed of beta-amyloid peptide, are the main signature of Alzheimer’s disease (AD). Two main forms of beta-amyloid in humans are 40 and 42-amino acid, long; the latter is considered more relevant to AD etiology. The concentration of soluble beta-amyloid-42 (Aβ42) in cerebrospinal fluid (CSF-Aβ42) and the density of amyloid depositions have a strong negative correlation. However, AD patients have lower CSF-Aβ42 levels compared to individuals with normal cognition (NC), even after accounting for this correlation. The goal of this study was to infer deviations of Aβ42 metabolism parameters that underlie this difference using data from the Alzheimer’s Disease Neuroimaging Initiative cohort. Aβ42 is released to the interstitial fluid (ISF) by cells and is removed by several processes. First, growth of insoluble fibrils by aggregation decreases the concentration of soluble beta-amyloid in the ISF. Second, Aβ42 is physically transferred from the brain to the CSF and removed with the CSF flow. Finally, there is an intratissue removal of Aβ42 ending in proteolysis, which can occur either in the ISF or inside the cells after the peptide is endocytosed. Unlike aggregation, which preserves the peptide in the brain, transfer to the CSF and intratissue proteolysis together represent amyloid removal. Using a kinetic model of Aβ42 turnover, we found that compared to NC subjects, AD patients had dramatically increased rates of amyloid removal. A group with late-onset mild cognitive impairment (LMCI) also exhibited a higher rate of amyloid removal; however, this was less pronounced than in the AD group. Estimated parameters in the early-onset MCI group did not differ significantly from those in the NC group. We hypothesize that increased amyloid removal is mediated by Aβ42 cellular uptake; this is because CSF flow is not increased in AD patients, while most proteases are intracellular. Aβ cytotoxicity depends on both the amount of beta-amyloid internalized by cells and its intracellular conversion into toxic products. We speculate that AD and LMCI are associated with increased cellular amyloid uptake, which leads to faster disease progression. The early-onset MCI (EMCI) patients do not differ from the NC participants in terms of cellular amyloid uptake. Therefore, EMCI may be mediated by the increased production of toxic amyloid metabolites.

## Introduction

### Biomarkers of beta-amyloid metabolism *in vivo* and their relevance to Alzheimer’s disease

Dr. Alois Alzheimer described a certain kind of dementia (later named after him [1]) as having specific histopathological correlates: senile plaques and neurofibrillary tangles [2]. Since then, it has been quite common to assume that the etiology and pathogenesis of this disease is associated with the main component of the extracellular deposits which is beta-amyloid protein (Aβ). Therefore, many clinical studies concentrated on the possibility of using available measures of Aβ presence for diagnostic purposes and predicting disease progression.

For most of the twentieth century, the brain could only be studied *postmortem*. With the increased sensitivity of analytical methods, measuring the concentration of various Aβ peptides (including 42 amino acids long peptide Aβ42 which is considered more relevant to the etiology of AD [3]) in cerebrospinal fluid (CSF) became possible [4, 5]. Then, positron emission tomography (PET)-measurable labels such as FDA-approved florbetapir and florbetaben, which bind to and accumulate in the senile plaques, became available [6]. It was found that patients with cognitive decline due to Alzheimer’s disease had significantly more dense amyloid deposits. The appearance of deposits was also shown to have a prognostic value as patients with amyloid deposits appeared much more prone to decline cognitively compared to amyloid-negative patients [7].

The concentration of beta-amyloid in CSF and the density of amyloid deposits are interconnected, as high levels of amyloid deposits are associated with significantly decreased Aβ42 levels in the CSF [8–10]. Due to this correlation, the overall diagnostic accuracy of CSF-Aβ42 and PET studies appears similar, while PET has higher specificity [6]. Despite their significant correlation, CSF-Aβ42 levels and PET imaging have independent predictive powers for different Alzheimer’s disease markers, such as AD diagnosis itself, hippocampal volume, and brain metabolism [9].

Based on an analysis of data from the Alzheimer’s Disease Neuroimaging Initiative (ADNI), Sturchio et al [11] reported that normal cognition in patients with brain amyloidosis is associated with higher CSF-Aβ42. In their study, amyloid PET-positive participants from the ADNI database, for whom a neuropsychological evaluation was performed, as well as a CSF specimen was collected within 1 year from a PET scan, were selected for analysis. The levels of soluble Aβ42 were statistically significantly lower in the following order: (Alzheimer’s disease, AD) < (mild cognitive impairment, MCI) < (normal cognition, NC). The same phenomenon was present in each group when amyloid-positive patients were separated into three tertiles (those with a high, medium and low amyloid load, respectively); patients with normal cognition had higher CSF-Aβ42 compared with patients with AD [11].

This observation can be deduced from and, thus, confirmed by the data of Mattsson et al. [9]. In their study, all available patients from the ADNI dataset were used. The correlation between CSF-Aβ42 and florbetapir PET with an AD diagnosis were estimated as the regression coefficients and compared between models, which considered the effect of each index either individually or combined. The model that used both indices as independent variables provided a statistically significantly better fit than models that considered either of the indices alone. The combination of CSF-Aβ42 and PET also provided a better prediction for cognitive assessment than the individual indices [9], as CSF-Aβ42 correlated with the cognitive status in individuals with the same amyloid load. In other words, for a given amyloid load, cognition indices correlate with CSF-Aβ42 levels. This correlation was also observed in patients with a positive amyloid load (the subset analyzed in the study by Sturchio et al. [11]).

Thus, CSF-Aβ42 concentration and beta-amyloid deposits are partially independent correlates of cognitive function [9]. More specifically, unlike PET, which positively correlates with AD diagnosis, the correlation between CSF-Aβ42 and an AD diagnosis or cognitive assessment is negative. Thereby, Mattsson et al. [9] essentially demonstrated that normal cognition is associated with higher CSF-Aβ42 levels. However, they did not specifically emphasize this fact and did not go further than stating that CSF-Aβ42 levels provide additional information independent of amyloid load measured by PET. In contrast, Sturchio et al. [11] attempted to interpret this fact by speculating that soluble amyloid has an unknown “good” function, so that insufficient soluble amyloid may be the reason for neuronal loss (as opposed to the idea that exceedingly high levels of amyloid deposits are “bad”). While this speculation sounds logical, it cannot explain why CSF-Aβ42 levels in patients with normal cognition but a high amyloid load can be the same as CSF-Aβ42 levels in patients with AD and low amyloid load [11].

To interpret the interactions between these two major biomarkers of AD, we estimated the parameters of beta-amyloid turnover by fitting a mathematical model to the data from the ADNI dataset. The modeling framework we used is typical for pharmacokinetic studies. Previously, a similar approach was used for studying the turnover of beta-amyloid in individual patients [12–16]. The experiments in these studies involved a stable isotope labeling kinetic (SILK) technique specially designed to allow collecting data on a timescale of hours [16, 17]. In our study, we applied statistical analysis to publicly available data acquired by routine methods from a large cohort of patients.

We considered that Aβ42 released by cells to the interstitial fluid (ISF) can be removed via several processes. First, Aβ42 is transferred to the CSF and is physically removed from the brain with the CSF flow. Second, soluble beta-amyloid aggregates and forms insoluble fibrils. This process is slow in the absence of aggregated peptide but accelerates after amyloid seeds are already formed. The aggregation process decreases the concentration of soluble beta-amyloid in the ISF, even though it does not remove the peptide from the brain. Finally, Aβ42 can be digested inside the brain through proteolytic degradation. The proteolysis can occur either in the ISF or inside the cells after the peptide is endocytosed by the cells. The SILK technique in its original form [16, 17] can be used to estimate the rate of irreversible loss [16] but is unable to decompose it into aggregation and intrabrain degradation components. The aim of this study was to test the hypothesis that AD patients have an increased aggregation-independent amyloid removal rate compared to cognitively normal individuals, which provides an important link between the products of beta-amyloid degradation and neurodegeneration (which ultimately leads to AD diagnosis).

## Methods

### Clinical dataset

We used non-personalized data obtained from the Alzheimer’s Disease Neuroimaging Initiative (ADNI) (http://adni.loni.usc.edu/). The ADNI was launched in 2003 as a public-private partnership, led by Principal Investigator Michael W. Weiner, MD. The primary goal of ADNI has been to test whether serial magnetic resonance imaging, positron emission tomography (PET), other biological markers, and clinical and neuropsychological assessments can be combined to measure the progression of mild cognitive impairment (MCI) and Alzheimer’s disease (AD). The study protocol for ADNI was approved by the local ethical committees of all participating institutions and all participants signed informed consent forms which included the consent for de-identified data being shared with the general scientific community for research purposes (https://adni.loni.usc.edu/wp-content/uploads/how_to_apply/ADNI_DSP_Policy.pdf). The authors were approved by ADNI for receiving and analyzing de-identified data. The manuscript was approved for publication in written in accordance with the policies of ADNI.

Our analysis included all ADNI participants for whom the ascertainment of normal cognition (NC), MCI, or AD, as well as a CSF collection, were made within one year from a PET scan identifying brain amyloidosis. The number of research subjects in the AD, NC, late-onset MCI (LMCI), and early-onset MCI (EMCI) groups was 143, 416, 340, and 476, respectively. All subjects were evaluated between June 2010 and February 2019. Amyloid positivity was defined by PET data according to ADNI guidelines as a standard uptake value ratio (SUVR) at or above 1.08 for [18]F-florbetaben or 1.11 for [18]F-florbetapir, with a higher SUVR indicating a greater amyloid plaque burden. Details regarding PET acquisition are described in previous publications and on the ADNI website (www.adni-info.org). Given the use of two different amyloid PET-tracers, SUVR levels were converted to centiloids (CL) using tracer-specific formulas provided by ADNI [18].

### The effect of CSF-Aβ42 levels on the fraction of subjects with AD in subpopulations with low and high densities of amyloid deposits

This part of the study only included AD patients and NC subjects who had an amyloid load exceeding 20 CL. In clinical settings, centiloid values > 20 CL indicated the presence of at least a moderate plaque density, while values above 50 CL best correlated with both neuropathological and clinicopathological diagnosis of Alzheimer’s disease [19]. All research subjects in this study were divided into two groups: those with a low load (20<CL<50) and those with a high load (CL≥50).

Within each group, we calculated the fraction of patients with an AD diagnosis as a function of CSF-Aβ42. To do this, we built a histogram using bins with widths of 500 pg/ml and left borders increasing from 50 pg/ml in increments of 50 pg/ml. The data for each patient could appear in multiple adjacent bins, and only bins that had more than 10 study subjects were used. We calculated the average CSF-Aβ42 and the fraction of AD patients in each bin. With the assumption that the fraction follows a binomial distribution, the standard deviation of the fraction of AD patients in each bin was calculated as $SD=\sqrt{P(1-P)/N}$, where *P* is the fraction and *N* is the total number of research subjects in the bin. Sigmoidal approximation was performed using the Solver module in MS Excel.

### The single compartment model of cerebral amyloid turnover

The model connects two major biomarkers of beta-amyloid turnover, CSF-Aβ42 and the density of deposits of aggregated beta-amyloid. CSF-Aβ42 was measured directly in the samples of the CSF taken by a lumbar puncture. PET is used to measure the density of amyloid deposits. The intravenously injected PET label crosses the blood-brain barrier and gets absorbed by the amyloid plaques. The PET signal from areas known to not accumulate plaques is subtracted from the total PET signal to characterize the density of amyloid deposits. The corrected PET signal obtained by this methodology was found to be proportional to the density of amyloid deposits [20, 21]. Therefore, we assumed the PET signal after conversion to centiloids as a measure of the density of plaques in CL units. Hereinafter, we refer to the units of PET signal (and, hence, the amyloid load) as CL.

The concentration of soluble amyloid in the interstitial fluid (ISF), which we denote as [*ISF*], is defined by several processes: 1) synthesis by cells, 2) filtration of the protein into the CSF, 3) aggregation into non-soluble plaques, and 4) intratissue removal (see Fig 2). The model is based on several assumptions:

1. Synthesis rate ($\tilde{SYN}$) is independent of both interstitial Aβ42 and the density of plaques.
2. The rate of removal of the protein through the CSF is a product of the CSF removal rate (*FLOW*_*CSF*_) and CSF-Aβ42 ([CSF]): *FLOW*_*CSF*_·[*CSF*].
3. The concentrations of the soluble beta-amyloid in the ISF and the CSF have a similar order of magnitude and are correlated [22, 23]. The model assumes a linear relationship between the concentrations of soluble Aβ42 in the ISF and the CSF with a coefficient of transfer *K*_*T*_: [*CSF*] = *K*_*T*_·[*ISF*].
4. Existing plaques serve as seeds for the aggregation of soluble Aβ42 in the ISF. The rate of loss of soluble Aβ42 in the ISF due to aggregation is the product of Aβ42 concentration in the ISF, the concentration of plaques ([*PET*], calculated from the intensity of the PET signal), and the coefficient of aggregation *K*_*a*_: *K*_*a*_·[*PET*]·[*ISF*].
5. The rate of intratissue removal of soluble Aβ42 from the ISF is proportional to the interstitial Aβ42 concentration, [*ISF*] with a coefficient of uptake *K*_*R*_: *K*_*R*_·[*ISF*].

We assume that at any given moment, [*ISF*] is at equilibrium:

$$\frac{d[ISF]}{dt}=\tilde{SYN}-{FLOW}_{CSF}\cdot [CSF]-K_{a}\cdot [PET]\cdot [ISF]-K_{R}\cdot [ISF]=0.$$

After substituting [*ISF*] with [*CSF*]/*K*_*T*_,

$$\tilde{SYN}-{FLOW}_{CSF}\cdot [CSF]-K_{a}\cdot [PET]\cdot \frac{\left[CSF\right]}{K_{T}}-K_{R}\cdot \frac{\left[CSF\right]}{K_{T}}=0.$$

By rearranging this equation, [*CSF*] can be expressed as a function of [*PET*]:

$$[CSF]=\frac{\tilde{SYN}\cdot K_{T}}{K_{a}\cdot [PET]+K_{T}\cdot {FLOW}_{CSF}+K_{R}}.$$

After introducing new parameters, $SYN=\tilde{SYN}\cdot K_{T}/K_{a}$ and *KF* = (*K*_*T*_·*FLOW*_*CSF*_+*K*_*R*_)/*K*_*a*_, the equation take the following form:

$$[CSF]=\frac{SYN}{[PET]+KF}.$$

The equation has two parameters, *SYN* and *KF*, which will be referred to as the amyloid synthesis rate and the amyloid removal rate, respectively, in the rest of this manuscript. Note that the removal rate *KF* is a sum of two terms: (*FLOW*_*CSF*_·*K*_*T*_/*K*_*a*_), which is proportional to the amyloid removal rate through the CSF, and the intratissue amyloid removal rate divided by the aggregation rate, (*K*_*R*_/*K*_*a*_).

We assume that the transfer coefficient *K*_*T*_ and aggregation rate *K*_*a*_ are invariant over the population, so these two components of the amyloid removal rate are defined by the rate of removal by CSF and intratissue removal, respectively. Units for all parameters and variables are provided in Table 1.

**Table 1:** Units for model variables and parameters.

Quantity	Units	Quantity	Units	Quantity	Units
*PET*	*CL*	$\tilde{SYN}$	*ng*·*ml*^−1^*s*^−1^	*K*_*R*_	*s* ^−1^
*CSF*	*ng*·*ml*^−1^	*FLOW*_*CSF*_	*s* ^−1^	*SYN*	*CL*·*ng*·*ml*^−1^
*ISF*	*ng*·*ml*^−1^	*K*_*a*_	*CL* ^−1^ *s* ^−1^	*KF*	*CL*

### Parameter inference

To infer the values of the parameters *SYN* and *KF* for each of the four groups (NC, EMCI, LMCI, and AD), we used the CSF-Aβ42 and the amyloid deposit density of each research subject (based on data provided by the ADNI). We assumed that the conditional probability distribution *p*([*CSF*]|[*PET*]) is log-normal. Therefore, the posterior probability density function (PDF) of the parameters can be represented as

$$p(SYN,KF,\sigma )∼\frac{1}{\sigma^{N}}\exp\left\{-\frac{1}{2\sigma^{2}}\sum_{i=1}^{N}r_{i}^{2}\right\},$$

where the residual $r_{i}=\ln{\left[CSF\right]}_{i}-\ln\left(SYN/({\left[PET\right]}_{i}+KF)\right),$ *σ* is an unknown standard deviation, *N* is the number of participants in the group, and ln(.) is the natural log function. To calculate the marginal PDF for *SYN* and *KF*, we integrated the posterior PDF over *σ* as follows.

$$\int_{0}^{\infty }\frac{1}{\sigma^{N}}\exp\{-\frac{1}{2\sigma^{2}}\sum_{i=1}^{N}r_{i}^{2}\}d\sigma =2^{k-1}\Gamma (k){\left(\sum_{i=1}^{N}r_{i}^{2}\right)}^{-k},$$

where *k* = (*N*−1)/2 and Γ(*k*) is the gamma function. Therefore, the following holds.


$$p(SYN,KF)=\int_{0}^{\infty }p(SYN,KF,\sigma )d\sigma ∼{\left(\sum_{i=1}^{N}r_{i}^{2}\right)}^{\frac{1-N}{2}}.$$

This marginal parameter distribution was used to calculate the confidence regions for the individual parameters on the (*SYN*, *KF*)-plane, as well as their standard errors.

### Calculations and statistical comparisons

Calculations were performed using MS Excel and custom written C++ programs. Comparison of parameter values between groups was performed using a z-test, where significance was defined as p<0.05. Values are presented as mean ± SEM.

## Results

### Patients with higher CSF-Aβ42 levels are less prone to AD

**Fig 1:**
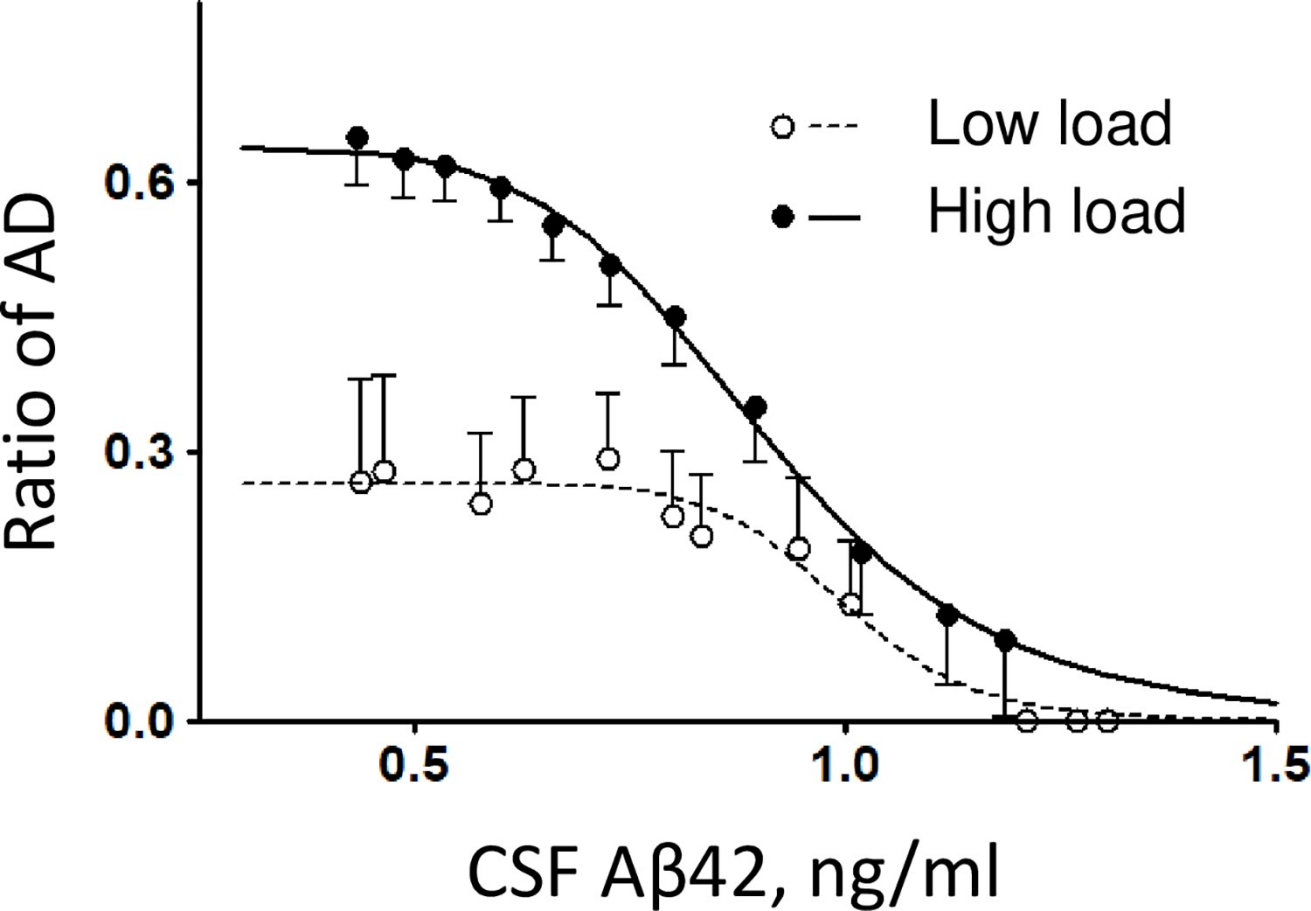
The relative frequency of AD patients as a function of the concentration of CSF-Aβ42 in subpopulations with low and high amyloid deposition density (amyloid load). Error bars indicate standard deviations. Lines represent the best fits by a sigmoid function.

The probability of an AD diagnosis in randomly selected research subjects from the study cohort clearly correlates with their CSF-Aβ42 levels. To illustrate this, we calculated the fraction of AD patients in two groups (both composed of the AD and NC participants) as a function of their CSF-Aβ42 levels (Fig 1). The first group included participants with a low amyloid load (see Methods), and the second group consisted of participants with a high amyloid load. The dependence appeared much more pronounced in research subjects with a high amyloid load—the fraction of AD patients increased from 10% at the highest observed CSF-Aβ42 values to 65% at the lowest observed CSF-Aβ42 values. Due to the small number of observations, we could not reliably calculate the percentage of patients with an AD diagnosis at the highest possible CSF-Aβ42 values, so we used a sigmoid approximation of the curve for extrapolation and found that the fraction of AD patients approached zero in subjects with both high and low amyloid load as CSF-Aβ42 exceeds 1 ng/ml. The highest percentage of patients with an AD diagnosis was observed when their CSF-Aβ42 levels were lowest. We estimated it to be 27% in subjects with a low amyloid deposit density and 65% in those with a high deposit density.

### The relationship between CSF-Aβ42 levels and amyloid load can be described using a simple mathematical model

**Fig 2:**
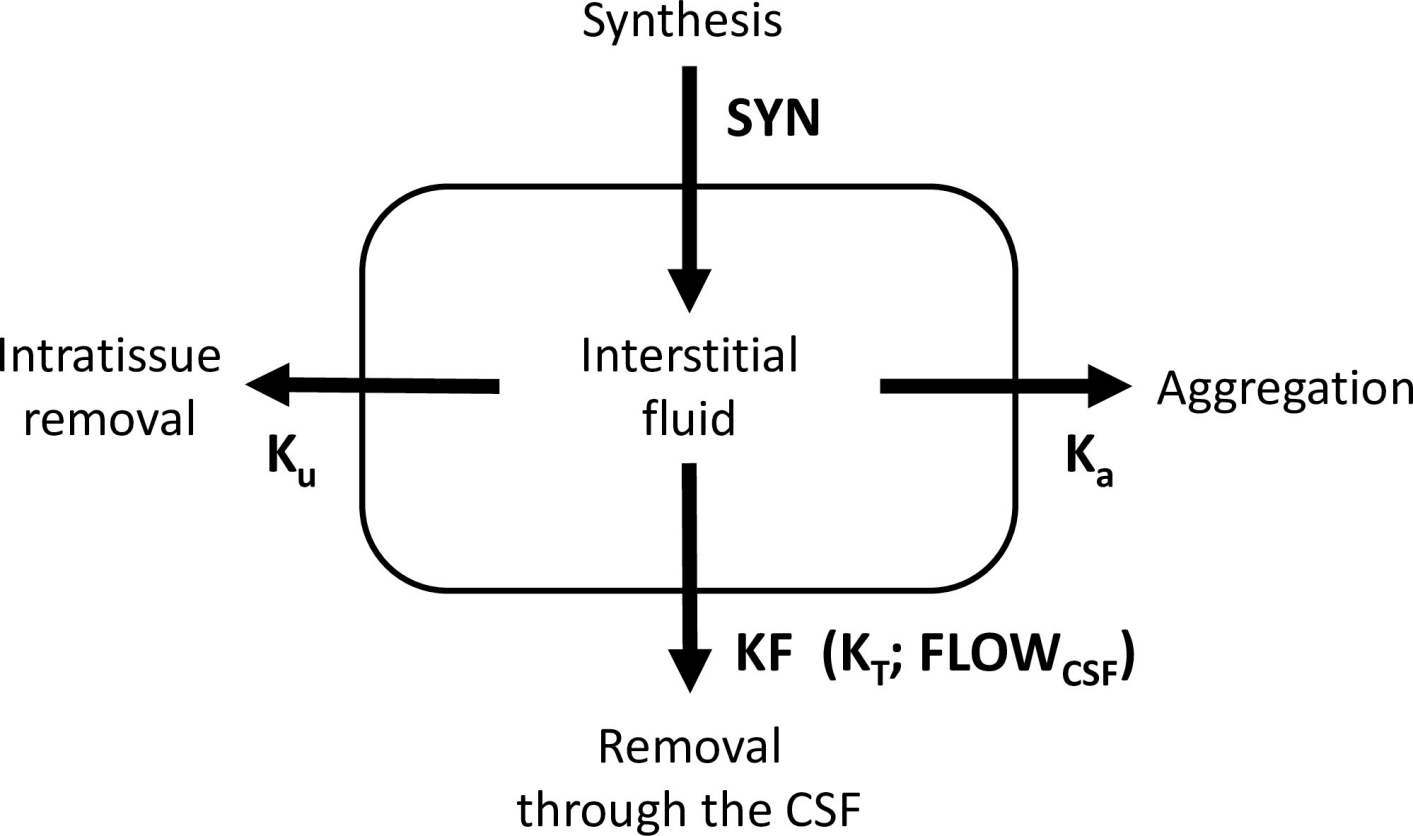
A schematic of the single compartment model of beta-amyloid turnover used to describe the mathematical relationship between CSF-Aβ42 and amyloid load in the brain. The parameters of the model are shown next to the arrows.

We used a single-compartment model to describe the beta-amyloid concentration in the interstitial fluid. The model considers Aβ synthesis by cells, its removal by the CSF, its cellular uptake by endocytosis, and its aggregation into amyloid deposits (Fig 2, see details in Methods). We found that the steady state of free amyloid concentration corresponds to a specific relationship between CSF-Aβ42 (denoted by [*CSF*]) and the density of amyloid deposits ([*PET*]):

$$[CSF]=\frac{SYN}{[PET]+KF}$$ (Eq 1)

where *SYN* is a parameter representing the synthesis rate and *KF* is a sum of the rate of amyloid removal through the CSF and the cellular amyloid uptake rate (see Methods for details). Eq (1) readily explains the negative correlation between CSF-Aβ42 and the density of amyloid deposits [24].

We then used the ADNI data set to infer the unknown values of the parameters by fitting the model to the data available for groups with different diagnoses under the assumption that CSF-Aβ42 is measured as a log-normally distributed quantity with unknown variance. Therefore, the statistical inference was performed based on the marginal probability density function (PDF) for *SYN* and *KF* obtained by the integration of the posterior PDF over the error variance (see Methods).

### AD patients have higher intratissue amyloid removal rate compared to subjects with normal cognition

**Fig 3:**
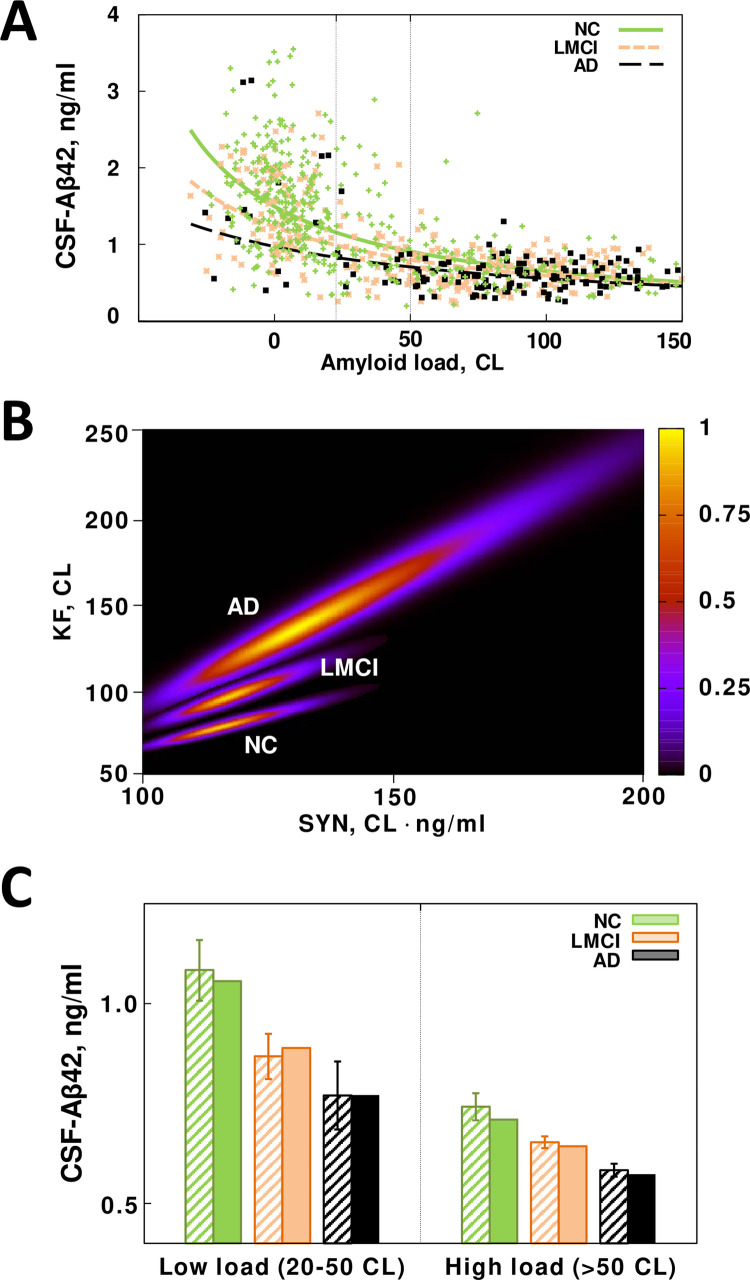
A comparison of beta-amyloid turnover parameters in subjects with normal cognition (NC), patients with Alzheimer’s disease (AD), and patients with late-onset mild cognitive impairment (LMCI). The parameters were inferred from two major AD biomarkers (CSF-Aβ42 and beta-amyloid density) in research subjects from the ADNI database. **A**. Scatter plot of CSF-Aβ42 vs beta-amyloid load for the three groups. Lines represent best fits by Eq (1) for each group. Vertical dotted lines show the range of the PET signal corresponding to the low amyloid load in panel C. **B**. Heat maps of the posterior probability distributions of the parameters characterizing beta-amyloid turnover in the three groups. **C**. Average CSF-Aβ42 in patients that have a low or high amyloid load. The values calculated using Eq (1) based on amyloid load with best-fit parameters (solid bars) are not statistically different from the values calculated using CSF-Aβ42 data explicitly (striped bars). The average CSF-Aβ42 in patients with a high amyloid load, regardless of their group, is significantly lower than the average in patients of the corresponding group that have a low load. CSF-Aβ42 is also progressively lower in patients with a more severe clinical condition, regardless of load. However, the average CSF-Aβ42 in subjects with normal cognition and a high load is not different from the average CSF-Aβ42 in patients with AD and a low load.

Fig 3A shows data on CSF-Aβ42 vs. PET-measured amyloid load in subjects with normal cognition (NC) as well as in patients with late-onset mild cognitive impairment (LMCI) or an AD diagnosis. The major difference between the clouds corresponding to the different groups is that on average AD patients tend to have greater amyloid loads compared to NC subjects. Patients with LMCI are more broadly distributed as this group includes both individuals with significant amyloid deposition (comparable to AD patients) as well as those who have low amyloid accumulation (similar to a majority of NC subjects). As far as CSF-Aβ42 levels are concerned, the shapes of the clouds are very similar for all three groups, and any differences in the relationship between CSF-Aβ42 and amyloid load are hardly distinguishable to the naked eye.

To elucidate these differences, we fitted the model described in the previous section to the data for each group (see also Methods) and calculated the PDF of the parameters linked to amyloid synthesis and removal, *SYN* and *KF* (see previous section). The three curves in Fig 3A correspond to the most probable values of the parameters in each group, revealing a tendency for CSF-Aβ42 to be progressively lower with an increasing severity of cognitive impairment (consistent with observations by Sturchio et al. [11]). To characterize the statistical significance of the changes in the parameter values, we calculated the marginal PDFs of the parameters, shown as heat map plots for each group in Fig 3B. The boundary between blue and black approximately corresponds to the boundary of the 95% confidence region. The regions appear not to overlap for the NC, LMCI and AD groups, indicating a statistically significant difference between the estimated parameter values. The difference was confirmed by a multi-variate z-test (p<0.05). The estimate of the amyloid removal rate appears to be higher in AD patients compared to NC subjects (see more about this comparison in the next section).

To illustrate that our model accurately predicts average CSF-Aβ42 based on amyloid load, we separated all amyloid-positive subjects in each group into those with low and high amyloid load (20<CL<50 and CL>50, respectively). For each research subject, we used either the measured CSF-Aβ42 value or the value calculated based on their [PET] and the model parameters for the corresponding group. The averages of the values calculated using the model (darker bars in Fig 3C) were not statistically significantly different from the averaged measured CSF-Aβ42 (lighter bars), confirming goodness of fit.

### Patients with early-onset mild cognitive impairment (EMCI) have parameters of Aβ42 turnover similar to those of NC subjects, unlike patients with late-onset mild cognitive impairment (LMCI)

**Fig 4:**
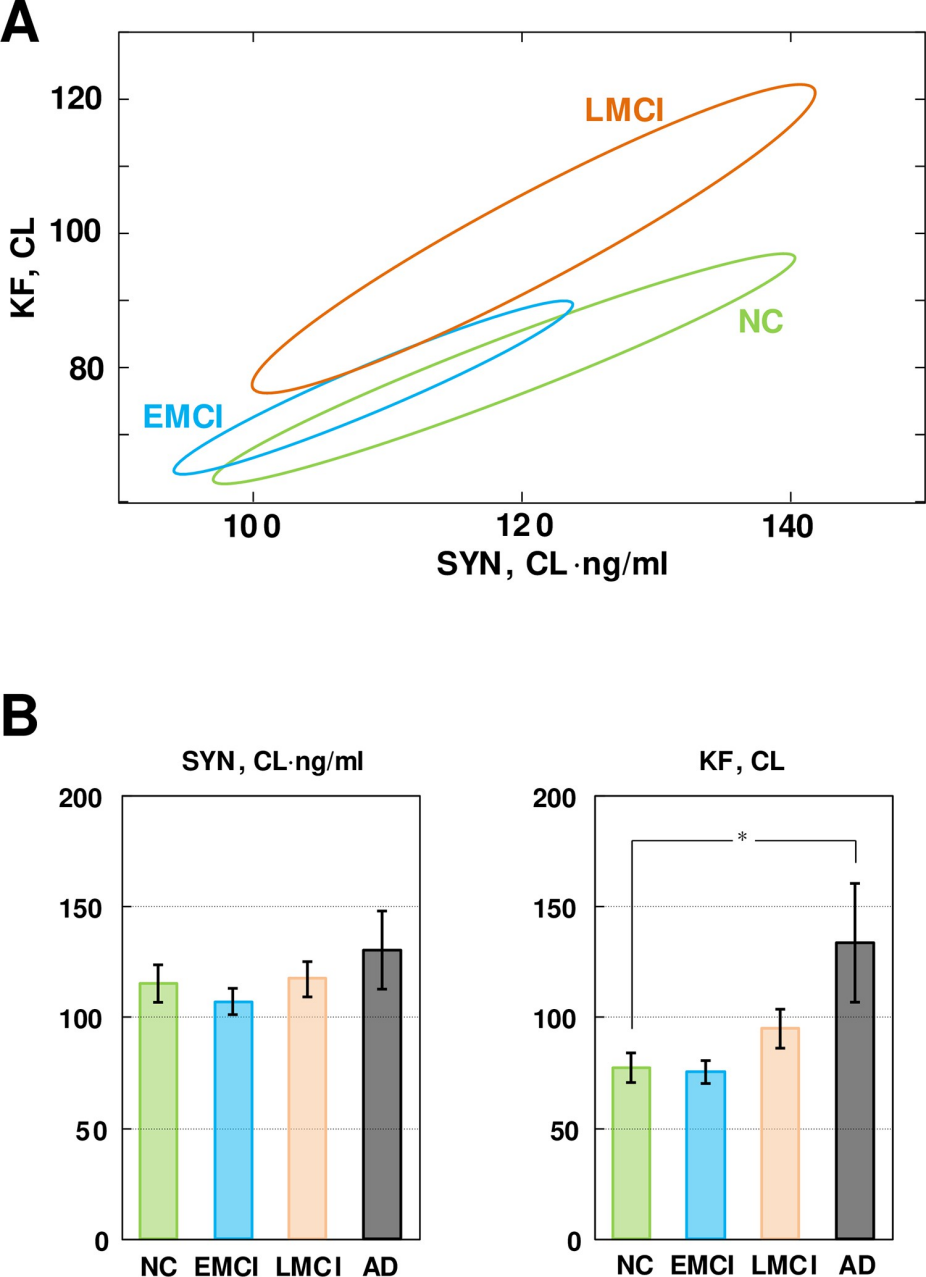
A comparison of beta-amyloid turnover parameters in subjects with normal cognition (NC), patients with either early-onset and late-onset mild cognitive impairment (EMCI and LMCI), and patients with Alzheimer’s disease (AD). **A**. The 95% confidence regions of the parameters characterizing beta-amyloid turnover in the NC, EMCI, and LMCI groups. The confidence regions for the NC and LMCI groups do not overlap, while the confidence regions for the NC and EMCI groups do. **B**. The inferred values of the beta-amyloid synthesis rate (SYN) and the removal rate (KF) for all studied groups. * The values of KF for the NC and AD groups are statistically different (z-test, p<0.05).

We extended our analysis by including the group of patients with EMCI whose data is also available in the ADNI database. Fig 4A shows 95% confidence regions of the model parameter estimates for the NC, EMCI, and LMCI groups. Patients with LMCI and NC subjects represent statistically different groups, whereas confidence regions for the EMCI and NC groups overlap. The latter suggests that EMCI patients may have beta-amyloid turnover parameters that are no different from those in NC subjects.

To characterize the effect of cognitive impairment severity on individual parameters, we also calculated marginal distributions for *SYN* and *KF*. Fig 4B shows the results in the form of a mean ± SD for each parameter in each group. Due to a significant correlation between the parameters (note the elongated shapes of the confidence regions in Figs 3B and 4A), the only significant difference detected was in the KF value between the NC and AD groups. Based on this, we conclude that AD patients have a significantly higher amyloid removal rate (KF) compared to cognitively normal participants. The difference between patients with LMCI and NC subjects is statistically significant only when both parameters, SYN and KF, are considered. The EMCI and NC groups do not appear to be significantly different in terms of these parameter values.

### The estimated intratissue beta-amyloid removal is dramatically higher in AD patients compared to NC subjects

Our calculations show that the amyloid removal rate, *KF*, was about 80 CL (centiloids) in the NC group, but 75% more (140 CL) in the AD group. As previously noted, our model does not allow for the independent estimation of the two components of *KF*–the rate of intratissue beta-amyloid removal and the rate of removal of beta-amyloid through CSF. As we speculate in the Discussion, however, the latter component is either not changed in AD patients [25] or decreased [26]. Therefore, the increase in the amyloid removal rate likely happens exclusively through an increase in intratissue beta-amyloid removal, and hence the increase of the intratissue removal rate in AD patients is likely to exceed 75%.

**Fig 5:**
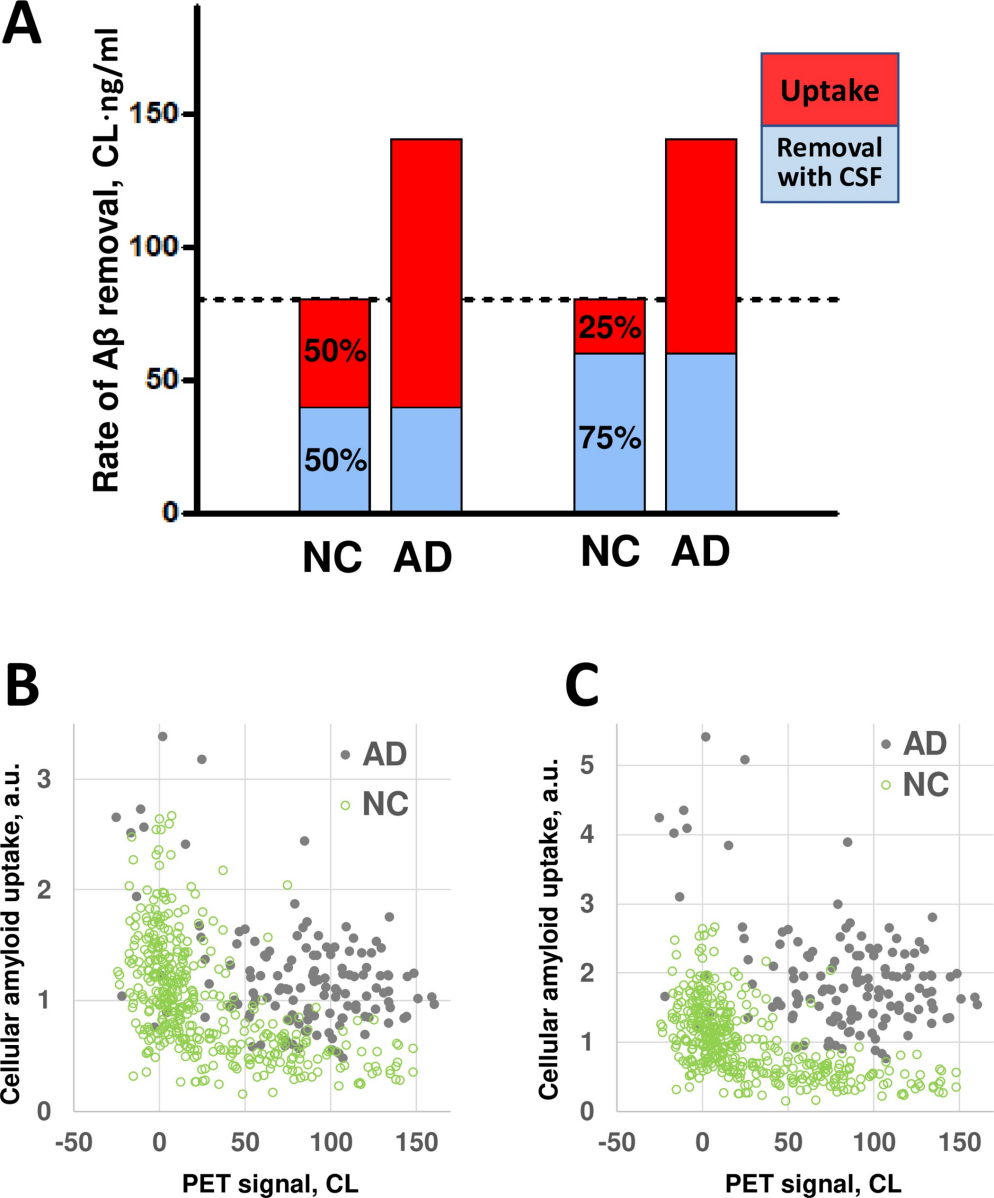
The difference in the aggregation-independent amyloid removal rate between NC participants and AD patients translates into a much greater relative difference in intratissue amyloid removal rate. **A**. The red and blue parts of the bars represent the intratissue amyloid removal rate and the rate of removal through the CSF (the components of the amyloid removal rate), respectively. If the ratio of the two components is 50/50 in the NC group, the intratissue removal rate is 2.5 times greater in the AD group than in the NC group. If the ratio is 75/25, the difference is 4-fold. **B**. The intensity of intratissue amyloid removal (in arbitrary units, a.u.) calculated for individual data points in the NC and AD groups. The rate of removal through the CSF is equal in both the NC and AD groups, and in this scenario is 50% of the amyloid removal rate of the NC group. **C**. The intensity of intratissue amyloid removal (in arbitrary units, a.u.) calculated for individual data points in the NC and AD groups. The rate of removal through the CSF is equal in both the NC and AD groups, and in this scenario is 75% of the amyloid removal rate of the NC group.

The estimate of a difference in the intratissue beta-amyloid removal rate between NC and AD subjects depends on the distribution of the amyloid removal rate between intratissue removal and removal through the CSF (see Methods). For the reasons outlined in the Discussion, it is likely that amyloid removal through the CSF dominates over intratissue removal. To examine possible scenarios, we considered intratissue removal to be 50% and 25% of the amyloid removal rate in NC subjects. To account for a 75% increase in the amyloid removal rate in AD patients, intratissue beta-amyloid removal rate should be increased by 2.5 and 4 times, respectively (Fig 5A).

Using this approach, it is possible to compare the intensity of the amyloid removal process as a product of the removal rate and the amyloid concentration in the CSF for individuals in the AD and NC groups from the ADNI cohort (Fig 5B and 5C). Again, we consider two scenarios where the intratissue removal rate constitutes 50% and 25% of the aggregation-independent removal rate in NC subjects. CSF-Aβ42 is on average slightly lower in AD patients (Fig 3C), but due to high subject-to-subject variability this effect is hardly seen at the individual level–CSF measurements heavily overlap for any given PET (Fig 3A). In contrast, the intensity of intratissue amyloid removal appears significantly higher in the AD group (Fig 5B and 5C). In the case of a 4-fold difference in the intratissue removal rate, the intratissue amyloid removal values for AD patients barely overlap with the intratissue amyloid removal values for NC subjects regardless of their amyloid load (Fig 5C).

## Discussion

### PET signal and CSF-Aβ42 are partially independent biomarkers

The brain amyloid load and CSF-Aβ42 have a strong negative correlation [8, 9, 24]. It is widely accepted that the negative correlation between amyloid plaque density and CSF-Aβ42 occurs because senile plaques create a sink for extracellular soluble Aβ42. In fact, the difference in CSF-Aβ42 between AD patients and NC subjects is in the range of 500 pg/ml [24], while the CSF flow for all subjects is approximately 20 ml/h [26]. The “sink” effect, therefore, should “preserve” approximately 10 ng/h, or close to 100 μg/year. The average extra Aβ42 load of the AD brain compared with a healthy brain is about 5 mg [27], which can indeed accumulate over 50 years. This makes the “sink” theory quite reasonable as an interpretation of the negative correlation between the CSF-Aβ42 and the PET signal. However, the analysis of the ADNI dataset performed by Mattson et al. [9] showed that these two parameters provided partially independent information, as autoregressive models that included both CSF-Aβ42 and PET measurements had better predictive power. Furthermore, using the same dataset Sturchio et al. [11] demonstrated that in the models adjusted for age, sex, education, APOE4, p-tau levels, and t-tau levels, lower CSF-Aβ42 levels are associated with a higher probability of AD and worse cognitive status, as well as various other indices of the disease even when the comparison was made between groups with the same brain amyloid load.

Our analysis of the ADNI dataset confirms that while the biomarkers have a strong negative correlation described by a single compartment model, CSF-Aβ42 in fact provides independent clinically valuable information. In cohorts with a similar amyloid load, a higher CSF-Aβ42 clearly correlates with a better clinical outcome. CSF-Aβ42 levels above 1000 pg/ml are associated with a low ratio of AD patients, even in the group with high amyloid loads (Fig 1). Lower CSF-Aβ42 corresponds to a greater increase in the fraction of AD patients if amyloid deposit density is high. Our results confirm and extend previously published analyses and suggest to consider both amyloid load and CSF-Aβ42 for more precise estimates of patient status and prognosis. It is clear that these two biomarkers together provide better insight into beta-amyloid metabolism in a particular patient.

### Intratissue amyloid removal rate may be dramatically higher in AD patients

Sturchio et al. [11] suggested that a high CSF-Aβ42 level plays a critical role in preserving proper brain function. Considering the challenges to this interpretation mentioned in the Introduction, we offer an alternative interpretation by analyzing the characteristic rates of amyloid turnover that we estimated from the same dataset. The model connecting CSF-Aβ42 to the density of amyloid deposits has several parameters that can all potentially be altered in AD. These parameters include the cellular amyloid synthesis rate, the rate of removal through the CSF, and the intratissue amyloid removal rate. Due to its biophysical nature, the aggregation rate is considered the same for all populations. Based on Eq (1), either the synthesis rate, *SYN*, should be lower, or the rate of removal, *KF*, should be higher, in order for the CSF-Aβ42 to be lower,. The suggestion of a lower synthesis rate in AD is not only counterintuitive, but is also not supported by experimental data [17]. With this in mind, the only remaining parameter involved in the turnover of Aβ42 that can explain the difference in CSF-Aβ42 levels between healthy and sick individuals is the amyloid removal rate. Our model predicts a 75% higher amyloid removal rate from the ISF in AD patients compared to cognitively normal individuals. As noted, amyloid removal occurs through both the CSF and cellular uptake, with the removal through the CSF having a greater share.

In scenarios where intratissue removal is between 50% and 25% of the beta-amyloid removal rate, a 75% increase in the aggregation-independent amyloid removal rate translates into a 2.5 to 4-fold increase in the intratissue amyloid removal rate. Furthermore, amyloid removal through CSF could be decreased in AD patients due to a lower CSF flow [26]. Therefore, to account for the estimated increase in the aggregation-independent amyloid removal rate, the intratissue removal has to increase even more strongly. We do not have data to estimate the upper boundary of this change, but even the lower boundary of a 2.5-fold increase in the intratissue amyloid removal rate is quite dramatic.

According to the model, the increased amyloid removal rate leads to a lower amyloid concentration in the CSF (see Eq (1)). The total amount of amyloid metabolized by the tissue is equal to the product of the intratissue removal rate and the amyloid concentration. However, the reduction in amyloid concentration in the CSF of AD patients compared to the concentration in healthy participants is only about 20–25%, while there is at least a 2.5-fold increase in the intratissue amyloid removal rate (Figs 3 and 4). Therefore, the amount of amyloid metabolized by the brain tissue can be more than two times greater in AD patients compared to cognitively normal individuals. As we will discuss below, this difference can be an etiology-based biomarker of AD.

### Increased cellular amyloid uptake may be one of the key molecular mechanisms defining the age-related progression of Alzheimer’s disease

The progression of Alzheimer’s disease is associated with neuronal death. The severity of the disease correlates with the density of beta-amyloid deposits. Although the aggregated amyloid is not toxic to cells, the synaptic loss and neuritic dystrophy are highest in anatomical proximity to the senile plaques [28]. On the other hand, it is well-established that soluble beta-amyloid is toxic to neurons both *in vitro* and *in vivo* [29–31]; however the mechanisms of its toxicity are still under debate [32, 33].

Recently, we introduced the amyloid degradation toxicity hypothesis, which explains known facts about beta-amyloid toxicity and suggests specific pathways leading to cell death [34]. According to the hypothesis, the formation of toxic amyloid products is initiated by the endocytosis of beta-amyloid and merging of the amyloid-laden endosomes with lysosomes. Intralysosomal digestion produces short fragments of the beta-amyloid peptide, which can create channels in the membranes of lysosomes [35–37]. These channels are non-selective and can be extremely large [35, 38–41, 35, 46]. Channel formation is frequently cited as a molecular mechanism underlying AD [42–47], but it is usually implied that the channels are formed in the plasma membrane, which does not explain many phenomena associated with the disease [48]. Our hypothesis suggests that amyloid membrane channels are formed in lysosomal membranes rather than in plasma membranes. Due to their composition, lysosomal membranes are a perfect target for membrane channel formation by beta-amyloid fragments [36]. The permeabilization of lysosomal membranes in cells exposed to beta-amyloid has been demonstrated by independent laboratories [49, 50]. It readily explains lysosomal/autophagy disfunction, which is considered a major feature of AD [51–53], as well as intracellular ion concentration perturbations induced by exposure to beta-amyloid [54]. Furthermore, lysosomal permeabilization can result in cell death through multiple mechanisms, which we have described previously [34].

The degradation of beta-amyloid is mediated by proteases [55]. However, most proteases are contained in lysosomes, so before being degraded, beta-amyloid must be endocytosed. An increased cellular uptake rate of beta-amyloid can explain the increased intratissue removal in our model. Per the amyloid degradation toxicity hypothesis, more intense amyloid endocytosis would result in a greater probability of lysosomal permeabilization and, subsequently, faster cell death (which is characteristic to AD). To be a pathophysiological factor of AD, the cellular beta-amyloid uptake should be increased before the development of cognitive deficiency. Therefore, if the measurement technique for the cellular amyloid uptake in patients is developed, not only can it be used for diagnostic purposes, but it could also predict the disease before any symptoms become evident.

The idea that neurotoxicity in AD is mediated by the cellular uptake of beta-amyloid could be key to understanding the non-effectiveness of the plaque dissolving approach in the treatment or prophylaxis of AD. According to our model, dissolving the aggregated beta-amyloid would decrease the removal of soluble beta-amyloid through aggregation and, thus, it would increase the interstitial Aβ concentration. In fact, the treatment with plaque-dissolving monoclonal antibodies does result in a significant increase of CSF-Aβ42 [56]. The increased availability of soluble peptide will increase the uptake and, correspondingly, increase neurotoxicity. Therefore, according to our model, dissolving the plaques can result in the acceleration of AD progression. One can argue that beta-amyloid deposits can lead to neuroinflammation by themselves or induce neurotoxicity in some other ways. In this case, the outcome of plaque dissolvement will depend on the balance between the effects promoting neurodegeneration (greater availability of soluble beta-amyloid) and the ones protecting from it (reduction of neurodegenerative action of amyloid fibrils). Such a competition may result in significant individual-to-individual variability, which is in line with recent large clinical trials for the effects of plaque-dissolving monoclonal antibodies [56].

### AD and EMCI are caused by different pathophysiological mechanisms

Our results suggest that AD and LMCI groups are characterized by an increased cellular amyloid uptake rate, which presumably results in an increased formation of toxic amyloid species and leads to irreversible neuronal damage. In contrast, beta-amyloid turnover in patients with EMCI does not appear different from that in NC subjects. Specifically, there is no tendency towards higher intratissue amyloid removal in EMCI patients (Fig 4B). Is it possible that beta-amyloid is more toxic in certain patients, even if their amyloid uptake rate is not elevated?

**Fig 6:**
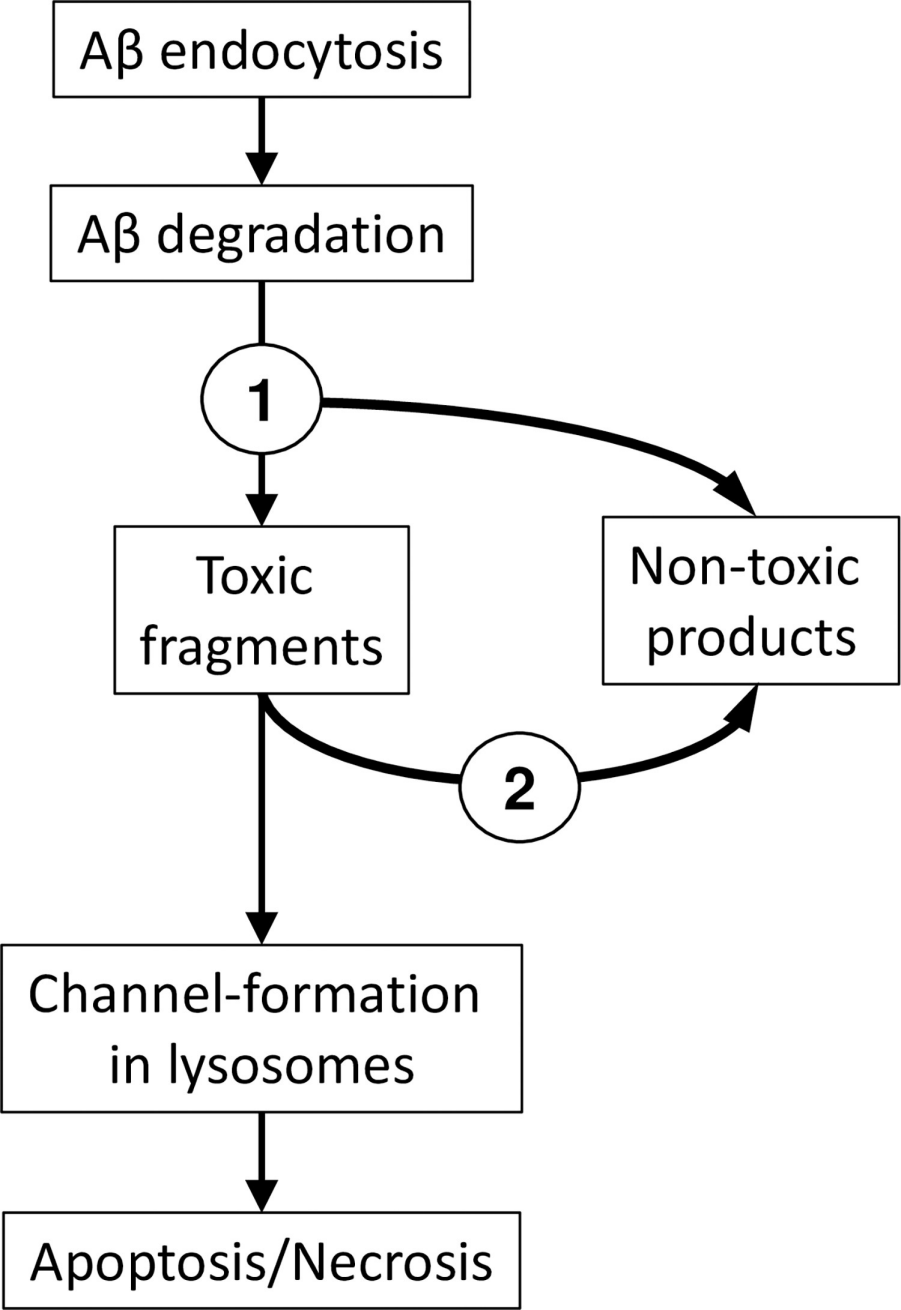
The sequence of events resulting in neuronal death and the progression of Alzheimer’s disease as suggested by the amyloid degradation toxicity hypothesis.

To answer this question, it is necessary to consider how amyloid toxicity develops at the cellular level. The production of peptide fragments is a part of lysosomes’ physiological function, which is to degrade large molecules such as proteins. Unlike short amyloid fragments, full-length Aβ42 is not able to form membrane channels in cell-like structures such as liposomes [36, 37]. Taken together, these facts allow us to hypothesize that the general sequence of intracellular events leading to cell death is as shown in Fig 6. Some beta-amyloid fragments are able to form membrane channels, and some are not [35–37, 46], so endocytosed beta-amyloid can be degraded by lysosomal proteases into both channel-forming and non-channel-forming fragments (Fig 6, dichotomy 1). In this context, the channel-forming ability of amyloid fragments is synonymous to their toxicity. Channel-forming fragments such as Aβ25–35, being relatively large peptides, are degraded further (Fig 6, dichotomy 2). Therefore, toxic amyloid degradation products have a limited life span, and membrane channel formation is most likely a relatively rare event [37]. However, once giant membrane channels are formed in lysosomes, they can leak lysosomal enzymes into the cytoplasm [48] which in turn leads to the activation of necrosis and/or apoptosis.

Based on the above, the rate of channel formation depends on the concentration of toxic fragments. This concentration, in turn, depends not only on the concentration of the full-length peptide but also on the balance between the formation and degradation of these fragments. Hypothetically, most toxic amyloid fragments are produced by endoproteases, while their degradation is performed by exoproteases. Therefore, the degradation is mediated by proteases that are not necessarily the same enzymes as the ones producing toxic amyloid fragments. A change in the balance between exo- and endoproteolytic activities of lysosomal proteases would affect the probability of membrane channel formation.

Thus, enzymatic disbalance (an increased rate of toxic fragment production and/or a slower degradation of toxic fragments) can be a mechanism for EMCI that is not associated with an increased cellular uptake rate of beta-amyloid. Alternatively, the concentration of toxic fragments can be increased due to a higher amyloid endocytosis rate, which appears to be the mechanism of AD and LMCI.

### Limitations and future directions

The single compartment model which we used in this study is oversimplified. First, we assumed that CSF-Aβ42 levels can be used as a surrogate for interstitial concentrations of Aβ42. This assumption is based on the correlation of the concentrations of the peptide in these two bioliquids [23], while there is in fact a complex process of amyloid exchange between the interstitial space and the CSF [57]. Second, the model assumes a homogeneous Aβ42 distribution within the interstitial compartment, however, different areas of the brain can have different synthesis and uptake rates of beta-amyloid. Nevertheless, the proposed model allows for the inference of the parameters characterizing beta-amyloid metabolism as an average over the entire brain. These findings add an organ system level into the integrative model of AD pathophysiology that we have previously proposed [34].

The precision of estimates is dependent on the number of observations, so increasing the size of the cohort can demonstrate statistical significance for the differences between the characteristic parameters of the groups. However, using a statistical approach does not allow for the estimation of the parameters of interest in specific patients. Establishing novel biomarkers, which directly characterize the rates of relevant processes such as cellular amyloid uptake and metabolic production of toxic amyloid species, can be a breakthrough for the early and/or differential diagnosis of Alzheimer’s disease.

## Conclusions

Based on this modeling study, we conclude that lower levels of soluble Aβ42 in CSF, considered in combination with the amyloid load, is an important biomarker suggesting an increased intratissue amyloid removal rate. We hypothesize that increased amyloid removal can be the consequence of increased cellular amyloid uptake in the brain, resulting in an increased production of neurotoxic amyloid degradation products. This suggests that the rate of beta-amyloid uptake can be tested as a novel pathophysiologically relevant biomarker of Alzheimer’s disease as well as a predictor of late-onset AD. A differential diagnosis of AD also requires an assessment of lysosomal proteolytic activity that can lead to the production of toxic amyloid fragments.

## Supporting information

S1 Data (XLSX)

## Abbreviations

AD: Alzheimer’s disease
MCI: mild cognitive impairment
LMCI: late-onset MCI
EMCI: early-onset MCI
NC: normal cognition
Aβ: beta-amyloid
Aβ42: Aβ_1–42_
CSF: cerebrospinal fluid
CSF- Aβ42: concentration of Aβ42 in the CSF
